# An update of stabilisation exercises for low back pain: a systematic review with meta-analysis

**DOI:** 10.1186/1471-2474-15-416

**Published:** 2014-12-09

**Authors:** Benjamin E Smith, Chris Littlewood, Stephen May

**Affiliations:** Physiotherapy Outpatients, London Road Community Hospital, Derby Hospitals NHS Foundation Trust, London Road, Derby, DE1 2QY UK; School of Health & Related Research, University of Sheffield, Regent Court, 30 Regent Street, Sheffield, S1 4DA UK; Faculty of Health & Wellbeing, Sheffield Hallam University, Sheffield, S10 2BP UK

**Keywords:** Systematic review, Meta-analysis, Low back pain, Exercise therapy, Core stability, Stabilisation, Treatment, Effectiveness

## Abstract

**Background:**

Non-specific low back pain (NSLBP) is a large and costly problem. It has a lifetime prevalence of 80% and results in high levels of healthcare cost. It is a major cause for long term sickness amongst the workforce and is associated with high levels of fear avoidance and kinesiophobia. Stabilisation (or ‘core stability’) exercises have been suggested to reduce symptoms of pain and disability and form an effective treatment. Despite it being the most commonly used form of physiotherapy treatment within the UK there is a lack of positive evidence to support its use. The aims of this systematic review update is to investigate the effectiveness of stabilisation exercises for the treatment of NSLBP, and compare any effectiveness to other forms of exercise.

**Methods:**

A systematic review published in 2008 was updated with a search of PubMed, CINAHL, AMED, Pedro and The Cochrane Library, October 2006 to October 2013. Two authors independently selected studies, and two authors independently extracted the data. Methodological quality was evaluated using the PEDro scale. Meta-analysis was carried out when appropriate.

**Results:**

29 studies were included: 22 studies (*n* = 2,258) provided post treatment effect on pain and 24 studies (*n* = 2,359) provided post treatment effect on disability. Pain and disability scores were transformed to a 0 to 100 scale. Meta-analysis showed significant benefit for stabilisation exercises versus any alternative treatment or control for long term pain and disability with mean difference of -6.39 (95% CI -10.14 to -2.65) and -3.92 (95% CI -7.25 to -0.59) respectively. The difference between groups was clinically insignificant. When compared with alternative forms of exercise, there was no statistical or clinically significant difference. Mean difference for pain was -3.06 (95% CI -6.74 to 0.63) and disability -1.89 (95% CI -5.10 to 1.33).

**Conclusion:**

There is strong evidence stabilisation exercises are not more effective than any other form of active exercise in the long term. The low levels of heterogeneity and large number of high methodological quality of available studies, at long term follow-up, strengthen our current findings, and further research is unlikely to considerably alter this conclusion.

**Electronic supplementary material:**

The online version of this article (doi:10.1186/1471-2474-15-416) contains supplementary material, which is available to authorized users.

## Background

Non-specific low back pain (LBP) can be described as low back pain without underlying cause or disease, and has a lifetime prevalence of 80% [1, 2]. Point prevalence ranges from 12% to 33%, with 90% of acute episodes recovering within six weeks [1, 3]. However, 62% of people experiencing their first episode of LBP will develop chronic symptoms lasting longer than one year, with 16% of people still sick listed from work at 6 months [4]. The UK health service spends more than £1 billion on related costs, including hospital and GP appointments and physiotherapy treatments, with similar high costs seen in other developed countries [5, 6]. LBP is a major cause for long term sickness amongst the workforce, and has been estimated to cost UK employers as much as £624 million per year, with 119 million works days lost each year [7, 8].

In the UK patients with LBP are routinely referred to physiotherapy [6]. Treatment can involve a number of different techniques ranging from spinal manipulations, mobilisation, advice, general exercises and specifically tailored exercises [9]. It has been claimed that there is a link between dysfunction within the activation and timing of local spinal stabilisation muscles and back pain [10–12]. Consequently a therapeutic exercise regime aimed at these muscles was developed, designed to ‘retrain’ motor skills and the activation dysfunction [12]. Despite doubts raised about this link between back pain and muscle activation, and the effectiveness of such an exercise regime (known as stabilisation or ‘core stability’ exercises) it has grown in popularity and now ranks the most common form of physiotherapy treatment in the UK for back pain [9, 13–15].

A 2008 systematic review by May and Johnson, which included 18 trials up to 2006, concluded that specific stabilisation exercises may be beneficial over no treatment, but went on to report that it was unlikely to produce an outcome better than any other form of exercise [13]. It has been suggested that median duration of survival time of a systematic review is 5.5 years, with 23% of systematic reviews being out of date within two years of publication [16]. Since 2006 there has been considerable growth in the evidence base, with a large number of new trials being published. In total there have been seven systematic reviews that have looked at stabilisation exercises [13, 17–22], with the previous three performing a meta-analysis [17, 21, 22]. Macedo et al [17] included studies published up to June 2008 and concluded that stabilisation exercises were no better than general exercise. In 2012 Wang et al [21] carried out a systematic review and also concluded there was no significant difference between ‘core stability’ and general exercises [21]. However, Wang et al’s narrow definition of ‘core stability’ exercises was “exercises performed on unstable surfaces”, rather than a broader definition based upon specific muscle activation. Furthermore, they only included randomised controlled trials (RCT) that specifically compared intervention versus general exercise, rather than any other alternative treatment, and only included people suffering back pain for more than three months. Consequently only five articles fulfilled their inclusion criteria [21]. Our systematic review uses a more broader definition and comparison, similar to May and Johnson [13], and found 19 further articles to add to the original 18 [13]. In contract to these results more recently Byström et al [22] reported that stabilisation exercises were more favourable than general exercises. They searched the literature up to October 2011, but did not limit their participants to non specific back pain and had far stricter inclusion criteria. Our review included a further 15 articles to Byström et al thus providing the justification for a more up to date review.

This systemic review and meta-analysis was conducted to update the 2008 data by May and Johnson [13]. The primary aim of this analysis is to systematically review the most current up to date literature to determine whether stabilisation (or ‘core stability’) exercises are an effective therapeutic treatment compared to an alternative treatment for people with non-specific low back pain. The secondary aim is to determine if stabilisation exercises are as effective as other forms of exercise, and to evaluate findings by meta-analysis if appropriate. This systematic review update followed the recommendations of the PRISMA statement [23].

## Methods

### Search strategy

An electronic database search of title and abstract was conducted October 2006 to October 2013 on the following databases: (1) PubMed, (2) the Cumulative Index to Nursing and Allied Health Literature (CINAHL), (3) The Allied and Complimentary Medicine Database (AMED), (4) Physiotherapy Evidence Database (Pedro), (5) The Cochrane Library. Specific search strategies depended on the particular database being searched. For the keywords and the PubMed search strategy used see Table 1. Hand searches of the reference list of included articles were also performed.

**Table 1:** **PubMed search strategy**

	Search term
1	Lumbar spine or back pain or low back pain
2	Randomized controlled trial or clinical trial
3	Exercises or rehabilitation or lumbar stabili* or core stability or transversus abdomin* or multifidus
4	1 and 2 and 3
5	Humans

### Study selection

For inclusion the studies had to meet the following criteria.

### Participants

Adults recruited from the general population with non-specific low back pain of any length of time. Low back pain defined as, but not restrictive to, pain and/or stiffness between the lower rib and buttock crease with or without leg pain. Studies with specific pathology, such as systemic inflammatory diseases, prolapsed disc, spondylolisthesis, pregnancy related, fractures, tumours, infections or osteoporosis were excluded.

### Interventions

Primary intervention arm of stabilisation, or ‘core stability’, exercises defined as: facilitation of deep muscles of the spine (primarily transversus abdominis or multifidus) at low level, integrated into exercise, progressing into functional activity, according to Richardson et al [12], Norris et al [24] or O’Sullivan et al [25]. Comparison group of any other intervention, placebo or control were considered appropriate.

### Outcomes

Included studies were required to report an outcome measure of pain and/or functional disability.

### Study design

Studies had to be full randomised controlled trials (RCTs), published in English, in a peer reviewed journal. Studies that were not randomised or quasi-random were excluded.

### Study selection

One reviewer (BS) conducted the electronic database searches and screened the title and abstracts. Full copies of potential eligible paper were retrieved and independently screened by two reviewers (BS and CL). Initial percentage agreement was 68%, and using Cohen’s statistic method kappa agreement was *k* = 0.29, which is considered poor to fair agreement [26–28]. Disagreements were resolved by consensus without the need for a third reviewer (SM), who was available. Initial disagreements were due to; intervention criteria [29–33], study population [34, 35], study design [36–39] and duplication of results from another publication being missed [40, 41].

Our review excluded participants with specific pathology, so all three reviewers (BS, CL and SM) verified any exclusion of studies from the 2008 review [13].

### Data extraction

We extracted the following data from the included articles: study design, participant information, interventions and setting, follow-up period and outcome data [42]. These data were then compiled into a standard table by one reviewer (BS) and then independently checked and verified by a second reviewer (SM). Disagreements were resolved through consensus. A third reviewer (CL) was available in the event of an agreement not being reached, but was not required. Of the included articles three had inconsistencies within their text, figures or tables with regards to their results [33, 43, 44]. All were contacted by e-mail, and all gave clarification. One study published median outcome scores, and the authors were contacted and provided mean outcome data [45]. Effectiveness was judged for short term (≤3 months from randomisation), medium term (>3 and <12 months) and long term (≥12 months), as recommended by the 2009 Updated Method Guidelines for Systematic Reviews in the Cochrane Back Review Group and in keeping with the original 2008 systematic review [13, 46].

Data from the 2008 review was taken directly from the published review [13].

### Quality assessment

Studies meeting the inclusion criteria were assessed for methodological quality and risk of bias using the PEDro scale [47]. The 11 item PEDro scale was developed by Verhagen et al using the Delphi consensus technique to develop a list of criteria thought by experts in the field to measure methodological quality [48]. The PEDro scale consists of the following items: (1) Was eligibility criteria specified? (2) Were all subjects randomly allocated? (3) Were allocations concealed? (4) Were the groups similar at baseline? (5) Was there blinding of all subjects? (6) Was there blinding of all therapists? (7) Was there blinding of all assessors? (8) Was there measures of at least one key outcome for more than 85% of the subjects initially allocated to groups? (9) Did all subjects for whom outcome measures were available receive the treatment or control condition as allocated or, where this was not the case, data for at least one key outcome was analysed by “intention to treat”? (10) Were the results of between group statistical comparisons reported for at least one key outcome? (11) Did the study have both point measures and measures of variability for at least one key outcome? [47]. Items 2 – 9 refer to the internal validity of a paper, and items 10 and 11 refer to the statistical analysis, ensuring sufficient data to enable appropriate interpretation of the results. Item 1 is related to the external validity and therefore not included in the total PEDro score [49].

All included articles were already scored within the PEDro database, and these data were extracted from the PEDro website [50]. Based upon the original 2008 paper and precedent within the literature, studies scoring ≥6 out of 10 were considered to be high quality [13, 51].

### Statistical analysis

Pain and disability mean scores, along with their measure of range (standard deviation/95% confidence interval) were transformed to a score ranging from 0 to 100 [52]. All data analyses were performed using the OpenMetaAnalyst software [53]. Statistical between study heterogeneity was assessed with the *I*^2^ statistic, and this review considered 25% low, 50% moderate and 75% high [54]. If trials were considered sufficiently homogenous then outcome data was pooled according to outcome (pain or disability), methodological quality (PEDro scores <6, or ≥6) and follow-up period. Due to the inherent heterogeneity in low back pain within the literature, the DerSimonian and Laird random effects model was used [55].

### Sensitivity analysis

The robustness of our results was tested through a sensitivity analysis. We assessed the impact of using solely high quality studies with using studies of low, medium and high quality combined.

## Results

### Study identification

**Figure 1:**
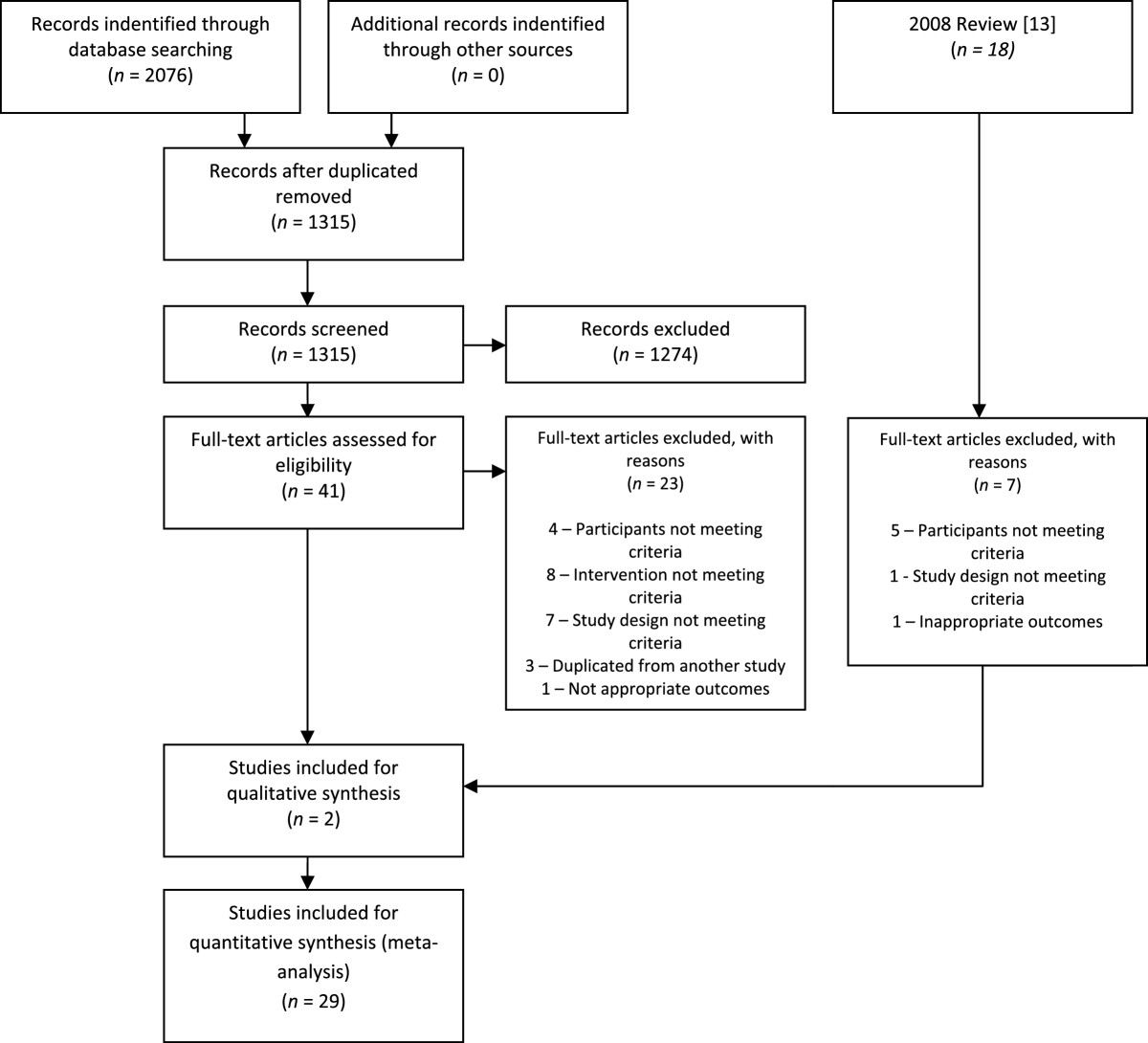
**Study selection process.**

The initial database search produced 2,076 citations, of which 41 were appropriate for full text review, see Figure 1 for study selection process.

After full text review 23 articles were excluded. Reasons being: participants not meeting criteria [34, 35, 56, 57], intervention not meeting criteria [29, 31, 32, 58–61], study design not meeting criteria [36–39, 62–64], duplications of results from other included studies [40, 41, 65] and no appropriate outcome measures [66]. That left a total 18 studies for inclusion [33, 43–45, 67–80]. Of the 18, two were separate publications of different treatment groups of the same larger study [71, 72]. Of note is that Franca et al [71] did not perform an intention to treat analysis, and so has a lower PEDro score than Franca at al [72]. However, as both had a PEDro score ≥ 6 this does not affect the pooling of both comparisons within the data synthesis. Therefore, a total of 17 separate trials were included.

From the 18 included studies from the 2008 review, seven were rejected for this review, five due to this review only including patients with non specific back pain [25, 81–84], one because it was a pilot study [85] and one due to inappropriate outcomes [86]. That resulted in 12 studies being drawn from the 2008 review (one from two publications) [87–99], with 29 studies in total included for this updated review.

### Characteristics of included studies

A summary of the characteristics of the included studies along with the main results is shown in Table 2. There were heterogeneous populations within the studies, with regard to duration of symptoms and presence of leg symptoms. Ten of the studies specified participants having back pain lasting more than three months [43, 67, 69, 71–73, 76–78], with two studies specifying pain lasting three or more months [68, 70], two specifying more than two months [33, 45], one specified any length of time [75], and two studies did not detail their criteria [44, 74]. Four studies included participants with or without leg pain [67–70], eight excluded participants with leg pain [33, 44, 45, 71–73, 77, 78] and six were not clear on their inclusion criteria with regards to leg pain [43, 74–76, 79, 80].

**Table 2:** **Characteristics of included studies**

Study characteristics	Participants characteristics	Intervention and setting	Outcome data/results
Akbari 2008 [67]	58 patients recruited from physiotherapy clinics in Iran (mean age = 39.8/sex not defined).	Physiotherapy clinic setting.	Main outcome assessed at baseline and at 8 weeks were:
		1. *N* = 29. Instruction of isometric abdominal drawing in manoeuvre, in 4 point kneeling, supine, sitting and standing. Progressing to 10 contractions of 10 second holds. Plus general exercises from group 2. 30 minute class, twice a week for 8 weeks.	1. Pain on visual analogue scale (VAS) (0–100).
2 Groups:			Both groups improved. Group 1 improved from mean pain scores of 7.25 (SD 0.97) to 2.5 (SD 1.24). Group 2 improved from 8 (SD 1.21) to 4 (SD 1.54).
	Inclusion criteria included:		
	a. Nonspecific LBP with or without leg pain		
1. General exercises plus motor control exercises	b. Pain > 3 month duration		There was no significance difference between groups (*P* = 0.83).
	c. Age > 18, < 80	2. *N* = 29. Exercises involving: lumbar flexion/extension in 4 point kneeling, mini sit up (head and shoulder off floor only), side plank, single leg extension holds in 4 pt kneeling and active prone lumbar extension. Hold times and repetitions based on clinical judgement. 30 minute class, twice a week for 8 weeks.	
2. General exercise			
Costa 2009 [68]	154 patients recruited from a physical therapy department of an Australian hospital (mean age = 53.7/79% male).	Hospital and home setting	Main outcomes assessed at baseline, 2, 6 and 12 months, include:
			1. Average numerical rating scale (NRS) for pain over last week (0–10)
2 Groups:		1. *N* = 77. 2 sessions a week in 1^st^ month, 1 session a week in 2^nd^ month, total of 12 hours, plus daily home based exercise. Stage 1: tailored exercises aimed at multifidus and transversus abdominis muscles. When the patient could achieve 10 repetitions of 10 second holds progressed to stage 2. Stage 2: Integrating contraction of muscles into more advanced function tasks and exercises, based upon individual therapy assessment.	
			2. Roland-Morris Disability Questionnaire (RMDQ) (0–24)
	Inclusion criteria included:		NRS baseline, 2, 6 and 12 months for group 1 was 6.8 (SD 2.1), 4.6 (SD 2.8), 5.0 (SD 2.9), and 5.0 (SD 2.9). Group 2 was 6.6 (SD 2.0), 5.6 (SD 2.6), 5.6 (SD 2.5) and 6.3 (SD 2.3).
	a. Nonspecific low back pain localized below the costal margin and above the inferior gluteal folds		
1. Motor control exercises			
2. Placebo of ultrasound therapy and detuned short-wave therapy	b. Pain ≥ 3 months duration		
	c. With or without radiculopathy		RMDQ baseline, 2, 6 and 12 months for group 1 was 13.1 (SD 5.0), 9.6 (SD 6.5), 10.3 (SD 7.0) and 11.4 (SD 7.8). Group 2 was 13.4 (SD 4.9), 11.9 (SD 5.9), 12.2 (SD 6.7) and 12.3 (SD 6.4).
	d. Age > 18, < 80		
		2. *N*=77. 20 minutesdetuned shortwave	
		diathermy and 5 minutes of detuned ultrasound for 12 sessions over 8 weeks.	No between group difference for pain at 2 and 6 months (*P* = .053 and *P* = 0.335). At 12 months group 1 better than 2 for pain by −1.0 (CI 95% -1.9 to −0.1) (*P*=0.030).
			Short term improvement in RMDQ at 2 and 6 months for group 1 compared to 2, (*P* = 0.003 and *P* = 0.014). But no difference at 12 months (*P* = 0.271).
Critchley 2007 [69]	212 patients recruited from primary or secondary care in a UK hospital (mean age = 44.3/36% male).	Hospital and home setting.	Main outcomes at 6, 12 and 18 months include:
			1. Roland-Morris Disability Questionnaire (RMDQ) (0–24)
3 Groups:	Inclusion criteria included:	1. *N*=71. Assessed and treated according to assessment findings. Treatments include combinations of joint mobilizations, manipulations, massage and exercise. Exercises included specific trunk muscle retraining, stretches, and general spinal mobility. Up to 12 sessions of around 30 minutes.	2. Pain on numerical analogue scale (0–100)
1. Usual physiotherapy			At 18 months RMDQ score improved from 11.1 (95% CI 9.6 –12.6) to 6.9 (95% CI 5.3– 8.4) with group 1, 12.8 (95% CI 11.4-14.2) to 6.8 (95% CI 4.9–8.6) with group 2, and 11.5 (95% CI 9.8 –13.1) to 6.5 (95% CI 4.5– 8.6) for group 2.
2. Spinal stabilization classes	a. Low back pain of more than 12 weeks duration		
3. Pain management classes	b. With or without leg pain		
	c. Age > 18		
		2. *N* = 72. Tailored to assessment findings and progressed within participants’ ability, working on transversus abdominis and lumbar multifidus muscle training followed by group exercises that challenged spinal stability. Maximum of 8 sessions of 90 minutes supervised exercise.	At 18 months pain improved from 60 (95% CI 54–66) to 39 (95% CI 31–46) for group 1, 67 (95% CI 61–73) to 32 (95% CI 24–40) for group 2 and 59 (95% CI 52–65) to 38 (95% CI 29–47) for group 3.
		3. *N* = 69. A combination of structured back pain education with group general strengthening, stretching, and light aerobic exercises. A maximum of 8 sessions of 90 minutes.	No between group differences were found for RMDQ (*P* =0.46) or pain.
Ferreira 2007 [70]	240 patients recruited from a physical therapy department from hospitals in Australia (mean age = 53.6/31% male).	Hospital and home setting.	Outcomes at 8 weeks, 6 and 12 months, include:
			1. Patient-Specific Functional Scale (PSFS) (3–30)
3 Groups:	Inclusion criteria included:	1. *N* = 80. Modelled on the ‘Back to Fitness’ program by Klaber	2. Roland-Morris Disability Questionnaire (RMDQ) (0–24)
1. General exercise		Moffet and Frost. 1 hour exercise group, 12 sessions over 8 weeks. Starts with 1 minute warm up followed by stretches and 10 exercises performed for 1 min each. Intensity dictated by patients’ response to exercise. Exercises include: walking/running on spot, sideways trunk curls, side steps/star jumps, press ups, side lying leg raises, prone leg raises, trunk curls, sit to stand, arm circling in 90° of abduction and bridging. Participants also encouraged to exercise at home at least once a day.	3. Average VAS (0–10) for pain over last week
2. Motor control exercise			
	a. Nonspecific low back pain with or without leg pain		Baseline, 8 weeks, 6 and 12 months scores for PSFS for group 1 were; 10.1 (SD 4.2), 14.4 (SD 6.6), 15.0 (SD 7.4) and 13.9 (SD 7.2). Group 2 were; 10.7 (SD 4.0), 17.7 (SD 6.2), 16.4 (SD 6.6) and 15.7). Group 3 were; 11.2 (SD 4.6), 17.5 (SD 6.8), 17.3 (SD 7.0) and 15.2 (SD 6.8).
3. Spinal manipulation	b. Pain ≥ 3 months duration		
	c. Age > 18, < 80		
		2. *N* = 80. Each patient was trained in contraction of transversus abdominis and multifidus muscles in isolation, by a physical therapist. Ultrasound was used to aid treatment when the therapist deemed appropriate. The contraction was then incorporated into more functional positions and tasks tailored to each patient. Patients were treated for 12 sessions over 8 weeks and encouraged to exercise at home daily.	Baseline, 8 weeks, 6 and 12 months scores for RMDQ for group 1 were; 14.1 (SD 5.5), 9.7 (SD 6.3), 10.1 (SD 7.0) and 9.6 (SD 6.9). Group 2 were; 14.0 (SD 5.3), 7.9 (SD 5.7), 8.4 (SD 6.4) and 8.8 (SD 6.5). Group 3 were; 12.4 (SD 5.7), 7.9 (SD 6.0), 7.7 (SD 6.3) and 9.2 (SD 6.6).
			Baseline, 8 weeks, 6 and 12 months scores for VAS for group 1 were; 6.5 (SD 2.1), 4.8 (SD 2.4), 4.8 (SD 2.6) and 5.2 (SD 2.8). Group 2 were 6.3 (SD 2.0), 4.0 (SD 2.5), 4.3 (SD 2.6) and 4.9 (SD 2.9). Group 3 were; 6.2 (SD 2.0), 4.1 (SD 2.6), 4.3 (SD 2.6) and 4.9 (SD 2.7)
		3. *N* = 80.Patiens were treated with spinal manipulations and mobilizations, at the discretion of the therapist, based on physical assessment. 12 sessions over 8 weeks and no exercises given.	
			At 8 weeks motor control and manipulation performed better than general exercise for PSFS. Adjusted mean difference in PSFS between group 2 and 1; 2.9 (95% CI: 0.9–4.8) (*p* = 0.004), between group 3 and 1; 2.3 (95% CI: 0.4–4.2) (*p* = 0.016).
			At 6 and 12 months all groups improved from baseline in all 3 outcomes. No apparent between group difference.
Franca 2010 [71]	30 patients recruited from an orthopaedic department in a Brazilian hospital (mean age = 42.0/26.7% men).	Hospital setting.	Main outcomes at 6 weeks:
			1. Visual Analogue Scale (VAS) (0-10cm)
2 Groups:	Inclusion criteria included:	1. *N*=15. 2 x 30 mins sessions a week for 6 weeks. Exercises focused on transversus abdominis and multifidus using the abdominal drawing in manoeuvre. Exercises in 4 point kneeling, crooked lying, prone and in upright positions.	2. Oswestry disability index (ODI) (%)
1. Segmental Stabilization			
			Both groups significantly improved from baseline. Group 1 improved in pain from 5.94 (SD 1.56) to 0.06 (SD 0.16) and ODI from 17.07 (SD 3.99) to 1.80 (SD 1.26). Group 2 improved pain from 6.49 (SD 1.48) to 2.89 (SD 1.45) and ODI from 17.27 (SD 3.84) to 8.40 (SD 3.13).
2. Superficial Strengthening	a. LBP > 3 months		
	b. Pain felt between T12 and the gluteal fold		
		2. *N* = 15. 2 x 30 mins sessions a week for 6 weeks. Exercises involving sit ups in crook lying, rotating sit ups, reverse sit up/hip flex in crook lying and prone trunk extension.	
			Group 1 produced significantly better results for pain and ODI than group 2.
Franca 2012 [72]	30 patients recruited from an orthopaedic department in a Brazilian hospital (mean age = 41.8/ sex not defined).	Hospital setting.	Main outcomes at 6 weeks:
			1. Visual Analogue Scale (VAS) (0-10cm)
2 Groups:	Inclusion criteria included:	1. *N* = 15. 2 x 30 mins sessions a week for 6 weeks. Exercises focused on transversus abdominis and multifidus using the abdominal drawing in manoeuvre. Exercises in 4 point kneeling, crooked lying, prone and in upright positions. 3 series of 15 repetitions of each exercise.	2. Oswestry disability index (ODI) (%)
1. Segmental Stabilization			Both groups significantly improved from baseline. Group 1 improved in pain from 5.94 (SD 1.56) to 0.06 (SD 0.16) and ODI from 17.07 (SD 3.99) to 1.80 (SD 1.26). Group 2 improved in pain from 6.35 (SD 1.51) to 3.15 (SD 1.20) and ODI from 18.73 (SD 3.61) to 9.20 (SD 4.09).
	a. LBP > 3 months		
2. Trunk and hamstring stretches	b. Pain felt between T12 and the gluteal fold		
		2. *N* = 15. 2 x 30 mins sessions a week for 6 weeks. Stretches involving knee hugs, hamstring stretches in supine, kneeling on heels and chest to thighs, global stretches of posterior muscles. 2 series of 4 minutes were performed, with 1 minute of resting interval.	
			Group 1 produced significantly better results for pain and ODI than group 2.
Gladwell 2006 [73]	49 patients from doctor surgeries and word of mouth in England, UK. (mean age = 42.1/22% male)	Home and class setting.	Main outcomes at base line and 6 weeks:
			1. Roland Morris pain rating visual analogue scale (RMVAS) (0–10)
2 Groups:	Inclusion criteria included:	1. *N*=24. Continued with normal activity and pain relief.	
1. Control of normal activity.		2. *N*=25. 6 x 1 hour Pilates class a week. Plus 2 x 30 minute sessions at home a week. Exercises involved initial teaching of recruitment of transversus abdominis, progressing onto recruitment during side plank, crook lying leg slides, bridging, supine leg lifts, 4 point kneeling leg extension slides, prone thoracic flexion, sitting lumbar flexion, sitting trunk rotation, crook lying arms circles, crook lying knee circles.	2. Oswestry disability index (ODI) (%)
2. Pilates	a. Pain located below the scapulas and above the cleft of the buttocks		
			Group 1, RMVAS changed from 2.4 (SD 0.9) at baseline to 2.4 (SD 0.8) at 6 weeks, and ODI improved from 24.1 (SD 13.4) at baseline to 18.1 (SD 13.0) at 6 weeks.
	b. Pain > 12 weeks		
	c. Age > 18, < 60		
			Group 2 improved RMVAS from 2.7 (SD 0.9) to 2.2 (SD 0.9) and ODI from 19.7 (SD 9.8) to 18.1 (SD 11.2).
			No significance difference between interventions was found.
Inani 2013 [74]	30 patients recruited from physiotherapy department of a hospital in India (mean age =30.4/66.7% male).	Department and home setting.	Main outcomes at baseline and at 3 months include:
			1. Oswestry disability index (ODI) (%)
2 Groups:	Inclusion criteria included:	1. *N* = 15. Short wave diathermy and lumbar traction. Teaching of isometric contraction of transversus abdominis and multifidus. Progressing onto contractions whilst holding static positions and progressing further into exercises, for example; hip flexion, extension, adduction and abduction in lying, side lying, standing or sitting. Duration of isometric holds, frequency and repetition not defined. Exact number of contact session also not defined.	2. Visual Analogue Scale (VAS) (0-10cm)
1. Core stabilization			Both groups significantly improved in outcomes. Group 1 ODI improved from 38 (SD 13) to 8.8 (SD 4.7) and VAS from 6.3 (SD 1.8) to 1.4 (SD 0.9). Group 2 improved ODI from 43 (SD 11) to 16 (SD 6.5) and VAS from 7 (SD 1.6) to 2.3 (SD 1.1).
2. Conventional exercises	a. Non specific low back pain		
	b. Age > 20, < 50		
		2. *N* = 15. Short wave diathermy and lumbar traction as group 1. General exercises include; general stretches, abdominal hollowing, isometric lumbar extension, bridges, graded flexion and extension exercises. Duration of holds, frequency and repetition not defined. Exact number of contact session also not defined.	There were significantly greater improvements in pain (*p* = 0.0018) and disability (*p* = 0.0309) for group 1 over 2
Javadian 2012 [43]	30 patients recruited in Iran, location not defined (age and sex not defined)	Class setting.	Main outcomes at baseline and at 3 months include:
2 Groups:	Inclusion criteria included:	1. N = 15. 15 minute warm up of cycling and general stretches of hip musculature, hamstring and calf. Stabilization exercises included isometric contraction of deep muscles of the lumbar spine in supine, bridging, kneeling, sitting and standing. Progressed onto Swiss ball and wobble board. Duration of isometric holds, frequency and repetition not defined. Routine exercises included single and double knee to chest, bridging, lower limb raises, supine cycling, heel slides, leg slides and crunches. Repetitions not defined.	1. Visual Analogue Scale (VAS) (0-100mm)
1. Stabilization exercises plus routine exercises			2. Oswestry disability index (ODI) (%)
	a. LBP > 3 months		Both groups significantly improved in outcomes. Group 1 improved in VAS from 45.06 (SD 4.15) to 18.41 (SD 2.15) and ODI from 43.84 (SD 5.55) to 16.83 (3.45). Group 2 improved in VAS from 47.73 (SD 3.82) to 9.58 (SD 1.56) and ODI from 45.80 (SD 6.64) to 5.16 (SD 2.16).
2. Routine exercises	b. Age > 18, < 45		
	c. At least 1 positive from the following:		
	1. Painful arc during flexion and return from flexion		
	2. Gower’s sign		
	3. Instability Catch		
			The control group improved more than the intervention group, but not significantly.
	d. Negative straight leg raise	2. N = 15. 15 minutes warm up as group 1, and routine exercises as group 1.	
	c. Positive prone instability test		
		Total number of classes over the 3 months not defined.	
Kumar 2010 [75]	141 patients recruited from a rehabilitation department of an India hospital (mean age = 35.1/64.5% male)	Rehabilitation department setting.	Main outcomes at baseline and 6 months.
			1. Visual Analogue Scale (VAS) (0-10cm)
		1. *N* = 69. Ultrasound 5 minutes, Short wave diathermy 15 minutes, plus lumbar strengthening exercises. These included 10 repetitions of; prone lying leg elevation, prone lying chest elevation and supine bridging. 20 sessions lasting approximately 40 minutes.	2. Quality of life health survey (SF-36) (36–151)
2 Groups:	Inclusion criteria included:		Group 1 improved in pain by 2.87 (SD 0.15) and group 2 improved by 3.95 (SD 0.26).
1. Conventional treatment			
	a. LBP of any duration		Group 1 improved in SF-36 by 10.70 (SD 5.9) and group 2 improved by 24.6 (SD 7.6).
2. Dynamic muscular stabilization techniques	b. Age > 20, < 40	2. *N* = 72. 20 sessions of one on one dynamic muscular stabilization exercise. Isometric abdominal drawing in manoeuvre in crook lying, progressing onto contraction holds with leg lifts. Progressing onto positions of sitting, 4 point kneeling, standing, supine and kneeling. Progressing onto functional high speed exercises, at the discretion of the therapist.	
		Patients were not allowed to have pain relief during the study period.	
Macedo 2012 [76]	172 patients were recruited by general practitioners or from a physical therapy department waiting list in Australia (mean age = 49.1/48.8% male).	Class and home setting.	Outcome taken at baseline, 2 months, 6 months and 12 months include:
		1. *N* = 86. 2 sessions a week in 1^st^ month, 1 session a week in 2^nd^ month, total of 12 hours, plus daily home based exercise. Plus 2 booster sessions at 4 and 10 months. Stage 1: tailored exercises aimed at multifidus and transversus abdominis muscles. When the patient could achieve 10 repetitions of 10 second holds progressed to stage 2. Stage 2: Integrating contraction of muscles into more advanced function tasks and exercises, based upon individual therapy assessment. Patients advised to do 30 minutes per week in 1^st^ month, and 60 minutes a week in 2^nd^ month.	
			1. Average numerical rating scale (NRS) for pain over last week (0–10)
2 Groups:			
			2. Roland-Morris Disability Questionnaire (RMDQ) (0–24)
1. Motor control exercises			Group 1 pain scores at baseline, 2, 6 and 12 months were 6.1 (SD 1.9), 4.1 (SD 2.5), 4.1 (SD 2.5) and 3.7 (SD 2.7).
	Inclusion criteria included:		
			Group 2 pain scores were; 6.1 (SD 2.1), 4.1 (SD 2.5), 4.1 (SD 2.7) and 3.7 (SD 2.6).
2. Graded activity	a. Chronic nonspecific low back pain		
	b. duration > 3 months		Group 1 RMDQ at baseline, 2, 6 and 12 months were; 11.4 (SD 4.8), 7.5 (SD 6.4), 8.0 (SD 7.1) and 7.4 (SD 6.7). Group 2 RMDQ were; 11.2 (SD 5.3), 8.0 (SD 6.5), 8.6 (SD 6.8) and 8.0 (SD 6.9).
	c. age > 18, < 80		
		2. *N* = 86. Same class duration, frequency and home exercises as group 1. The programme included individualized and submaximal exercises working on generalized (whole body) exercises without consideration of specific muscle activity. It was aimed at ignoring illness behaviours and reinforcing wellness behaviours. Cognitive behavioural principles were used to help the participants overcome the natural anxiety associated with pain and activities.	
			There were no significant differences between treatment groups at any of the time points.
Marshall 2013 [77]	64 patients were recruited via community advertising in Australia (mean age = 36.2/62.5% male).	Exercise class setting.	Outcomes taken at baseline, 2 months and 6 months include:
			1. Visual Analogue Scale (VAS) (0-10cm)
2 Groups:		1. *N* = 32. 50–60 minutes, 3 x a week for 8 weeks. The teaching of the isometric abdominal drawing in manoeuvre, with biofeedback pressure transducer under lumbar spine. Working in side lying, prone lying positions with upper and lower limb exercises. Including warm and cool down with whole body stretches.	
			2. Oswestry disability index (ODI) (%)
			VAS for group 1 at baseline was 3.6 (SD 2.1). Difference at 2 and 6 months from baseline were; −1.9 (CI 95% −2.6 to −1.2) and −1.6 (CI 95% -14.2 to −6.7). VAS for group 2 at baseline was 4.5 (SD 2.5). Difference at 2 and 6 months from baseline were; − 0.8 (CI 95% −1.5 to −0.1) and −1.2 (CI 95% −1.9 to −0.6).
1. Specific trunk exercises	Inclusion criteria included:		
2. Stationary cycling	a. Pain between the costal margins and inferior gluteal folds		
	b. Age > 18, < 50	2. *N* = 32. 50–60 minutes, 3 x a week for 8 weeks. Stationary bike, with variation in seated/standing positions, resistance and cadence with warm up and down and whole-body stretching.	
			ODI for group 1 at baseline was 25.4 (SD 11.2). Difference at 2 and 6 months from baseline were; −10.4 (CI 95% −14.2 to −6.7) and −10.4 (CI 95% −14.0 to −6.8). ODI for group 2 at baseline was 24.0 (SD 11.9). Difference at 2 and 6 months from baseline were; −3.9 (CI 95% −7.8 to 0) and −5.9 (CI 95% −9.5 to −2.4).
	c. Duration > 3 months		
			VAS significantly lower at 2 months for group 1 (*p*<0.05). ODI scores also significantly lower in group 1 at 2 months (*p*=0.019). Between group differences were no longer observed at 6 months.
Moon 2013 [78]	21 patients recruited from a rehabilitation outpatient clinic in Korea (mean age = 28.5/66.7% male).	Class setting.	Outcomes taken at baseline at and 8 weeks include:
2 Groups:			1. Visual Analogue Scale (VAS) (0–100)
	Inclusion criteria included:	1. *N*=11. 60 minute class, twice a week for 8 weeks. 15 minute warm up of stretches and 10 minute warm down, same in both groups. All exercises were performed with the abdominal drawing in manoeuvre and included crook lying, knee lifts, leg slides, straight leg raises, plank, 4 point kneeling leg lifts and alternate leg and arm lifts, bridging on Swiss ball, sitting on chair and Swiss ball, prone lying, side plank, wall sits with Swiss ball, 4 point kneeling pelvic tilts, sit up with Swiss ball (number of exercises and duration of hold not defined).	
			2. Oswestry disability index (ODI) (%)
1. Stabilization exercise			
			Group 1 baseline pain score was 33.5 (SD 18.4) and group 2 was 34.2 (SD 17.1). Baseline ODI group 1 was 14.7 (SD 2.9) and group 2 15.5 (SD 4.3)
2. Dynamic strengthening exercises	a. Non specific LBP.		
	b. Pain > 3 months		
	c. Without nerve root pain		At 8 weeks group 1 improved in VAS by
			16.7 (SD 7.0) and group 2 by 14.1 (SD 8.2). Group 1 improved ODI by 6.1 (SD 1.9) and group 2 by 3.6 (SD 1.5).
		2. *N* = 10. 60 minute class, twice a week for 8 weeks. 15 minute warm up of stretches and 10 minute warm down, same in both groups. Exercises performed included crook lying, bridging, mini sit ups, sit ups, twisting sit ups, knee to chest, prone leg extension, prone spine extensions, 4 point kneeling leg lifts, and alternate leg and arm lifts (number of exercises and duration of hold not defined).	No significant difference between groups at 8 weeks for pain (*p*=0.66) or ODI (*p*=0.07).
Rasmussen-Barr 2009 [45]	71 patients recruited from a private outpatient physiotherapy clinic in Sweden (mean age = 38.5/49.3% male).	Class and home setting.	Main outcomes taken at baseline, 8 weeks, 6, 12 and 36 months include:
		1. *N* = 36. Weekly exercise class for 8 weeks, lasting 45 minutes. Treatment included instruction on deep muscles of lumbar spine and isometric contraction of transversus abdominis with and multifidus by the abdominal drawing in manoeuvre. Bio-feedback was used. The class progressed to performing the exercise in different postural positions. Progression was based upon pain response (exact postural positions, duration of hold and repetitions not defined). Patients encourage of perform exercise at home daily for 15 minutes.	
2 Groups:			1. Oswestry disability index (ODI) (%)
	Inclusion criteria included:		2. Visual Analogue Scale (VAS) (0–100)
1. Graded exercise			
			Group 1 baseline ODI score was 20 (25th/75th percentiles 12/26). Changes in score compared to baseline at 8 weeks, 6, 12 and 36 months were −7 (−15/-4), −9 (−19/-2), −10 (−20/-2) and −11 (−23/-4) respectively.
2. Daily walks	a. Mechanically induced LBP		
	b. > 8 weeks duration		
			Group 2 baseline ODI score was 22 (14/28) and changes in score compared to baseline at 8 weeks, 6, 12 and 36 months were −4 (−10/0), −4 (−10/0), −2 (−12/2) and −6 (−14/0) respectively.
	c. ≥ 1 pain free period in last year		
		2. *N* = 35. Patients had 1 x 45 minute appointment at the start of the 8 weeks, and at the end. Instructed to take 30 minutes of walking a day at fastest pace possible without increasing pain (2 x 15 minutes allowed). Also a general home exercise plan provided, with no follow up instruction provided (exact exercises not defined).	
	d. Pain below the costal margin and above the inferior gluteal folds.		
			Group 1 baseline VAS score was 32 (25th/75th percentiles 18/59). Changes in score compared to baseline at 8 weeks, 6, 12 and 36 months were −15 (−31/-8), −15.5 (−30/-3.5), −12 (−34.5/-3) and −14 (−40/-4.5) respectively.
	e. No leg pain		
	f. Working		Group 2 baseline VAS score was 38 (23/62) and changes in score compared to baseline at 8 weeks, 6, 12 and 36 months were −8 (−19/-1), −9 (−24/0), −12 (−22/0) and −12 (−23/-2) respectively.
	g. Age > 18, < 60		
			Significant improvement in ODI with group 1 over group 2 (*p*=0.003). No between groups difference in pain was found.
Rhee 2012 [44]	42 patients recruited in Seoul, Korea (mean age = 50.2/50% male).	Class and home setting.	Main outcomes taken at baseline and at 4 weeks include:
			1. Million Visual Analogue Scale (MVAS) (0–150)
		1. *N* = 21. Supervised spinal stabilization exercises 3 times a week over a 4-week period, plus 5 x a week home exercises. Exercise involved abdominal drawing in manoeuvre in 5 different positions; prone, prone with leg and arm lifts, 4 point kneeling leg and arm lifts, crook lying mini sit ups, crook lying mini twist sit ups. Duration of hold and repetitions not defined.	2. Oswestry disability index (ODI) (%)
2 Groups:	Inclusion criteria included:		Both groups significantly improved for MVAS score (*p*<0.01). Group 1 improved from 42.70 (SD 13.80) to 32.81 (SD 10.85). Group 2 improved from 33.26 (SD 15.27) to 23.42 (SD 13.43)
1. Spinal stabilization exercises	a. Recurrent LBP		
2. Control	b. At least 1 previous episode		
			Both groups significantly improved for ODI score (*p*<0.001). Group 1 improved from 27.76 (SD 12.11) to 25.29 (SD 12.59) and group 2 improved from 17.29 (SD 9.15) to 12.52 (SD 8.50).
	c. Age > 21		
	d. No leg pain		
		2. *N* = 21. Provided with an education/advice booklet.	
Sung 2013 [33]	46 patients recruited in Cleveland, Ohio (mean age 50.4/47.8% male).	Class and home setting.	Main outcomes taken as baseline and at 4 weeks include:
			1. Oswestry disability index (ODI) (%)
2 Groups:		1. *N* = 21. Class setting for 20 minutes, 1 x a week for 4 weeks. Plus at home daily for 20 minutes. Exercise involved abdominal drawing in manoeuvre in 5 different positions; prone, prone with leg and arm lifts, 4 point kneeling leg and arm lifts, crook lying mini sit ups, crook lying mini twist sit ups. 5 second holds 2 x 15 reps.	Group 1 significantly improved from 24.89 (SD 11.89) to 17.73 (SD 11.75) (*p* = 0.03). Group 2 improved from 26.69 (SD 8.65) to 24.46 (SD 8.87) (*p* = 0.40).
	Inclusion criteria included:		
1. Core stabilization exercise	a. LBP > 2 months		
2. Spinal flexibility exercise	b. Age > 21		
			No statistically significant difference between groups.
	c. No leg pain	2. *N* = 25. Class setting for 20 minutes, 1 x a week for 4 weeks. Plus at home daily for 20 minutes. Exercise involved; single and double knee to chest in supine, 4 point kneeling flexion stretch, sitting flexion stretch and standing side flexion stretch. 5 second holds 2 x 15 reps.	
Unsgaard-Tondel 2010 [79]	109 patients recruited from general practitioners, physical therapist and advertisements at a hospital in Norway (mean age = 40.0/30.3% male).	Local fitness centre, physical therapy department of hospital and home setting.	Main outcomes taken at baseline, 8 weeks and 1 year include:
3 Groups:			1. Numerical rating scale (NRS) for current pain (0–10)
			2. Oswestry disability index (ODI) (%)
		1. *N*=36. 1 x 40 minutes one on one treatment at physical therapy department for 8 weeks. Teaching of isometric contraction of transversus abdominis with the use of ultrasound machine for feedback, initially in crook lying. Progression of exercises were individualized, but incorporated the isometric contraction of the local muscles. Participants were encouraged to perform the exercises at home, 10 contractions of 10 second holds 2 – 3 x a day.	NRS for group 1 at baseline, 8 weeks and 1 year was; 3.31 (SD 1.42), 1.76 (SD 1.54) and 2.01 (SD 1.94). Group 2; 3.61 (SD 1.75), 2.34 (SD 2.26) and 2.70 (SD 2.22). Group 3; 3.30 (SD 1.74), 2.73 (SD 2.32) and 2.66 (SD 2.03).
1. Motor control exercises	Inclusion criteria included:		
2. Sling exercises			
	a. LBP > 3 months		
3. General exercises	b. Age > 19, < 60		
	c. Pain > 2, < 10 Numeric Pain Rating Scale (NPRS) (0–10		ODI for group 1 at baseline, 8 weeks was; 19.44 (SD 8.38) and 12.78 (SD 7.62). Group 2; 22.28 (SD 11.22) and 16.18 (SD 10.88). Group 3; 20.84 (SD 9.43) and 17.75 (SD 9.63).
		2. *N*=36. 1 x 40 minutes one on one treatment at physical therapy department for 8 weeks. Unloading elastic bands were attached to the pelvis to help participants maintain the neutral spine position through a range of leg and arm positions and movements. Progression of exercises were individualized, but generally was achieved through reducing the elastic band support. The number of repetitions and sets was individualized.	
			No between group difference for pain 8 weeks (*p*=0.19) or 1 year p = 0.42) and no between group difference for ODI at 8 week (*p* = 0.21)
		3. *N*=37. Local fitness centre classes of 2 – 8 patients for 1 hour, 1 x a week for 8 weeks. General trunk strengthening and stretching exercises. For example resisted trunk flexion, extension and rotation. 10 repetitions, in 3 sets.	
Wang 2012 [80]	60 patients recruited from an outpatient rehabilitation department in a Chinese hospital (mean age = 38.6/58.3% male).	Class setting in rehabilitation centre.	Main outcomes taken at baseline and at 12 weeks include:
			1. Visual Analogue Scale (VAS) (0–10)
2 Groups:		1. *N* = 32. 40 minutes, 3 x a week for 12 weeks. 5 minute warm up. 30 minutes of exercises including; control of neutral spine alignment in sitting, prone, bridging, leg lifts, double knee flexion and reverse bridge. 5 minute warm down. Increasing difficulty as appropriate (exact details of exercises and progression not defined).	
1. Core stability exercises	Inclusion criteria included:		2. Oswestry disability index (ODI) (%)
2. Control of conventional exercise			VAS improved in group 1 from 5.52 (SD 3.46) to 2.15 (SD 1.58) and in group 2 from 5.11 (SD 2.78) to 2.92 (2.13).
	a. LBP > 3 months		
	b. Age > 19, < 60		
	c. Pain reproduced by movement		ODI improved in group 1 from 33.11 (SD 5.73) to 15.34 (SD 7.65), and in group 2 from 30.42 (SD 7.44) to 19.18 (SD 10.21).
		2. *N* = 28. 40 minutes, 3 x a week for 12 weeks. 5 minute warm up. 30 minutes of exercises including; sit ups, straight leg raises, bilateral straight leg raises, prone trunk extension. Then 5 minute warm down. Gradually increasing difficulty over 12 weeks (exact details of exercises and progression not defined).	
			Significant difference in favour of group 1 for VAS (*p* = 0.036) and ODI (*p* = 0.027) at 8 weeks.

Stabilisation exercises were the sole intervention for the majority of the studies, with five being individually treated [68, 70–72, 75, 79] and nine being in a class setting [33, 44, 45, 64, 73, 76–78, 80]. Three studies combined stabilisation exercises with other forms of treatment, such as general exercises [43, 67], and electrotherapy treatment [74].

Thirteen studies used a visual analogue scale to measure pain [43–45, 67, 70–75, 77, 78, 80], whilst four used an ordinal numerical rating scale [62, 63, 70, 73]. Four studies used the Roland-Morris disability Questionnaire (RMDQ) to measure disability [62, 63, 70, 73], whilst 12 measured disability using the Oswestry Disability Index (ODI) [33, 43–45, 64, 71–74, 77–80]. Two studies also included the Fear-Avoidance Beliefs Questionnaire (FABQ) as an outcome measure [77, 79].

Sixteen studies recorded short term follow-up, with 14 measuring pain and disability [43–45, 68, 70–74, 76–80], one just pain [67] and one just disability [33]. Seven studies recorded medium term follow-up, with six recording outcomes for pain and disability [45, 68–70, 76, 77], and one just pain [75]. Six studies recorded long term follow-up, with five recording pain and disability [45, 68–70, 76], and one just pain [79]. Two further studies went on to record follow-up of disability and pain extra long term [45, 69].

For the characteristics of the 12 included studies from the 2008 review, please refer to the original review [13].

### Study quality and bias

The PEDro scores ranged from 4 to 9 [47], with mean score of 6.6 (please refer to the PEDro website for score breakdowns). All participants were randomly allocated and all studies provided adequate results and analysis (items 10 and 11). Only five studies failed to conceal allocation [43, 67, 73, 74, 78] and one study assess baseline comparability [75]. No study blinded therapists, and only three blinded their participants [68, 75, 80]. The lower scoring studies were mainly marked down on blinding of assessors, adequate follow-up, intention to treat analysis and concealed allocation. With all studies, the greatest possible source of bias was related to blinding. Eleven publications scored ≥6 [33, 45, 68–72, 76–80], along with seven from the 2008 review, totalling 18 studies of high quality [88–91, 93, 95, 98].

### Data synthesis

Four studies from the 2008 review had insufficient data to enable their inclusion into a meta-analysis [89, 92, 94, 96], one of which was a high quality paper [89]. Twenty-two studies remained, 17 of high quality, which were considered suitably similar to warrant quantitative analysis and synthesis. Too few studies (only two of high quality) provided data ≥18 months to warrant pooling of data results for extra long term.

### Pain

**Figure 2:**
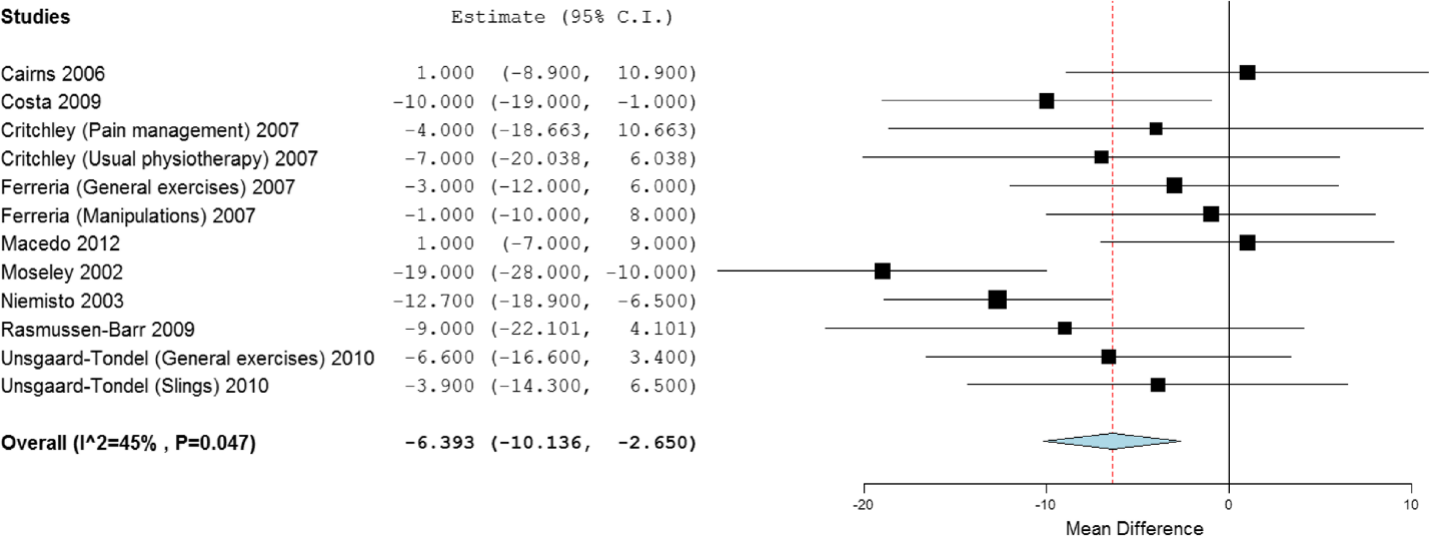
**Forest plot of stabilisation versus alternative intervention: pain - long term.** *Negative values favour stabilisation intervention, positive favour control.

Twenty-two studies, with 2,258 participants, provided post treatment effect on pain. Combining the results of high quality studies demonstrated significant benefit (mean difference) of stabilisation exercises for low back pain short, medium and long term of -7.93 (95% CI -11.74 to -4.12), -6.10 (95% CI -10.54 to -1.65) and -6.39 (95% CI -10.14 to -2.65) (Figure 2) respectively, when compared with any alternative treatment or control. However, the difference between groups was clinically insignificant with Minimal Clinical Important Difference (MCID) for pain being suggested as 24 to 40 [100], with between study heterogeneity high to moderate (*I*^2^ = 67%, 50% and 45% respectively).

**Figure 3:**
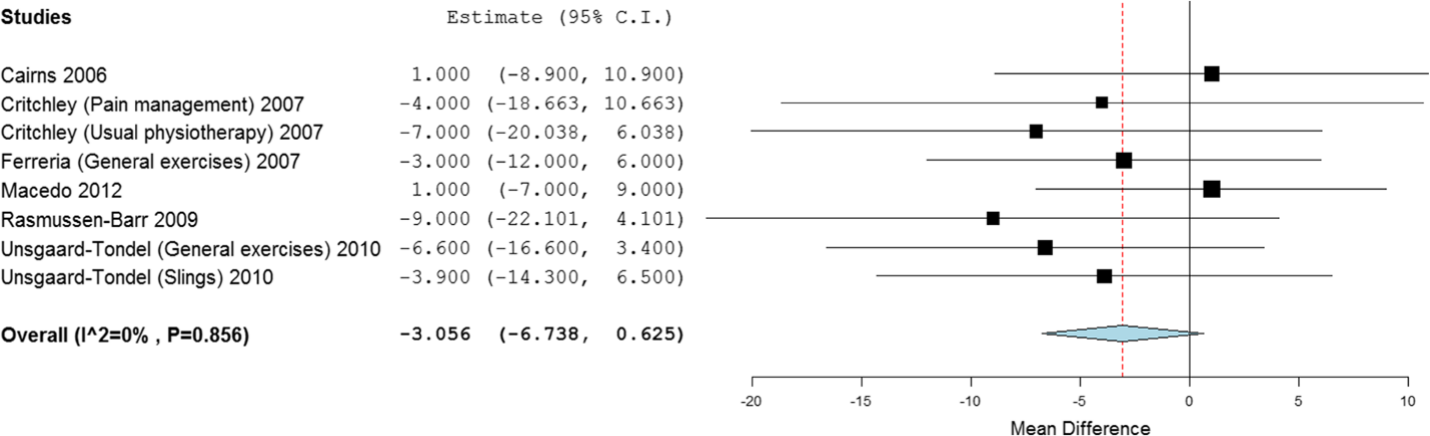
**Forest plot of stabilisation versus other exercises: pain - long term.** *Negative values favour stabilisation intervention, positive favour control.

Subgroup analysis of stabilisation exercises versus other forms of exercise demonstrated statistical significant short and medium term benefit, with a mean difference of -7.75 (95% CI -12.23 to -3.27) and -4.24 (95% CI -8.27 to -0.21). Differences between groups was clinically insignificant [100]. At long term there was no statistical or clinically significant difference; -3.06 (95% CI -6.74 to 0.63) (Figure 3). Between study heterogeneity was high to negligible (*I*^2^ = 66%, 0% and 0% respectively).

Combining the results of all studies for the sensitivity analysis provided very similar results (Additional file 1).

### Disability

**Figure 4:**
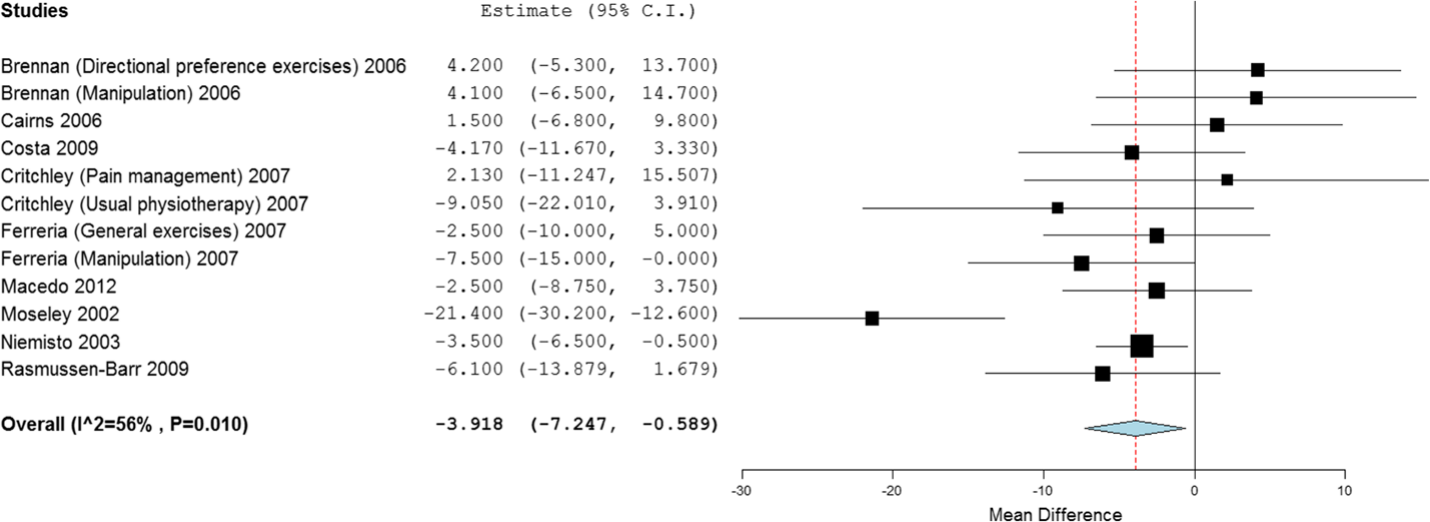
**Forest plot of stabilisation versus alternative intervention: disability - long term.** *Negative values favour stabilisation intervention, positive favour control.

Twenty-four studies, with 2,359 participants, provided post treatment effect on disability. Combining the results of high quality studies demonstrated statistical significant benefit (mean difference) of stabilisation exercises for low back pain short and long term of -3.61 (95% CI -6.53 to -0.70), -3.92 (95% CI -7.25 to -0.59) (Figure 4), when compared with any alternative treatment or control. However, the difference between groups was clinically insignificant, with MCID for RMDQ 17 to 21 and 8 to 17 for ODI (converting all to 0 – 100 scale) [100]. There was no difference statistically or clinically medium term; -2.31 (95% CI -5.85 to 1.23). Between study heterogeneity was high to moderate (*I*^2^ = 83%, 65% and 56% respectively).

**Figure 5:**
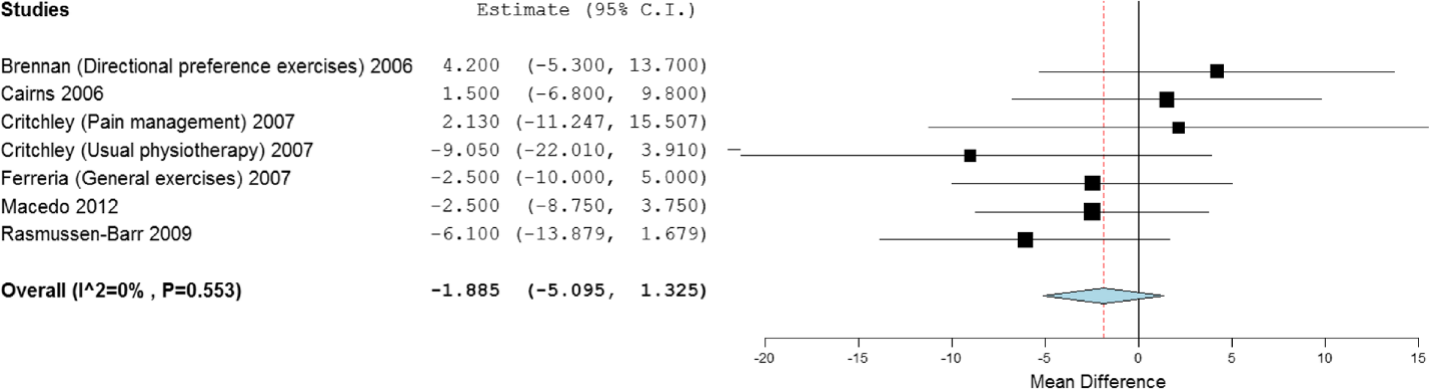
**Forest plot of stabilisation versus other exercises: disability - long term.** *Negative values favour stabilisation intervention, positive favour control.

Subgroup analysis of stabilisation exercises versus other forms of exercises demonstrated significant short and medium term statistical benefit, but no significant clinical difference, (mean difference) of -3.63 (95% CI -6.69 to -0.58) and -3.56 (95% CI -6.47 to -0.66). There was no significant statistical or clinical long term benefit; -1.89 (95% CI -5.10 to 1.33) Figure 5. Between study heterogeneity was high to negligible (*I*^2^ = 82%, 0% and 0% respectively).

Combining the results of all studies for the sensitivity analysis provided results that were less favourable for stabilisation exercises for short to medium term, with similar long term results (Additional file 1).

Two high quality studies featured FABQ as an outcome measure. FABQ (physical activity) (0-24) and FABQ (work) (0-42) for Marshall and Kennedy [77] at short term follow-up had a non significant mean difference of 2.2 (95% CI −1.3 to 5.6) and 2.3 (95% CI −1.8 to 6.5) respectively in favour of stabilisation exercises, when compared to stationary bike exercises. There was a non significant medium term mean difference of −2.0 (95% CI −5.1 to 1.0) and −2.7 (95% CI −7.6 to 2.1) respectively in favour of the stationary bike. Short term mean difference for FABQ (physical activity) for Unsgaard-Tøndel et al [79] was non significant at -1.58 (-4.00 to 0.84) and -0.18 (-2.42 to 2.07) in favour of sling and general exercises, respectively. Mean difference for FABQ (work) was non significant at -0.40 (95% CI -3.81 to 3.01) in favour of slings and 0.25 (95% CI -2.74 to 3.24) in favour of stabilisation exercises, when compared to general exercises.

## Discussion

### Summary of main findings

The objective of this systematic review was to evaluate the current evidence for the benefit of stabilisation (or ‘core stability’) exercises for low back pain. The overall results of the meta-analysis indicates a trend favouring core stability exercises which is not regarded as clinically significant, when compared with any alternative treatment or control. Minimal clinical important difference (MCID) for pain has been suggested as 24 to 40, with 17 to 21 for RMDQ and 8 to 17 for ODI (converting all to 0 – 100 scale) [100]. Any reduction in favour of stabilisation exercises was potentially meaningless, with mean change scores for pain (7.93, 6.10 and 6.39) and disability (3.61, 2.31 and 3.92) falling well below these MCID levels.

The overall results of the subgroup meta-analysis suggest that stabilisation (or ‘core stability’) exercises for low back pain offer very minimal benefit in the short and medium term when compared with other forms of exercise, with mean change scores for pain (7.75 and 4.24) and disability (3.63 and 3.56) also falling well below the clinically significant level. There was no significant benefit in the long term, for pain or disability, when compared with any other form of exercise. Results were trending towards stabilisations, but results were not significant, and any benefit would be clinically insignificant, being largely below the MCID level.

In the subgroup analysis of long term follow-up for stabilisations exercises versus other forms of exercises heterogeneity was negligible (*I*^2^ = 0%). Therefore, our results, that stabilisation exercises offer no benefit over alternative forms of exercises in the long term, can be considered robust.

Whilst not statistically significant, both studies that used FABQ as an outcome found that there was a trend of worse scores with stabilisation exercises, compared with stationary bikes, sling exercises and general exercises [77, 79]. The rehabilitation strategy surrounding stabilisation exercises has been challenged and has been suggested could encourage unhealthy thoughts and beliefs on pain and movement [101].

### Limitations of included studies

For the meta-analysis of pain and disability for stabilisation versus any alternative treatment or placebo, high to moderate heterogeneity existed. *I*^2^ scores of pain for short, medium and long term were 67%, 50% and 45%, and disability, 83%, 65% and 56% respectively. The high heterogeneity is possibly due to the different comparisons being made between trials, and this reduces the robustness of our short to medium term results. Overall, the interventions were applied to a wide variety of patients, including patients from low, medium or high socio-economic groups, unemployed or employed, having had investigations or no investigations, patients with or without leg pain, patients with acute or chronic symptoms and patients classed as ‘distressed’ or ‘distressed’ patients excluded. Patients that have high levels of fear avoidance scores are likely to have poor outcomes and compliance with biomedical models of pain and treatments, such as stabilisation exercises, and would likely do better with a biopsychosocial approach [101]. Cairns et al. [88], for example, excluded patients that were ‘distressed’, which perhaps biases results in favour of stabilisation exercises. This compares with Ferreira et al [70], whose participants were from low socio-economic groups, who are more likely to develop chronic pain states with worse outcomes, and would perhaps bias results in favour of alternative treatment protocols [102]. Furthermore, differences existed with how the treatments were delivered, class settings only, one to one treatment only, class/one to one treatment with home exercises or just home exercises, plus different amount of therapist contact times.

The studies included within the main meta-analysis had PEDro scores of ≥6, and as such were considered to have low bias. However, the main source of bias within the studies was blinding. No study blinded the therapist and few studies blinded the participants. Given that the pain and disability rating scales were patient self recorded it is possible that this could over estimate the treatment effect sizes. However blinding in active physiotherapy studies is difficult to achieve.

One of the limitations with long term follow-up of RCTs, particularly with exercise intervention, is the attrition rate. An uneven dropout has the risk of over estimating the effect size of treatment groups. For example Ferreira et al. [70] had an uneven dropout rate, with 9% for the general exercise group and 19% for the stabilisation group. This could easily bias the results in favour of the stabilisation group.

### Limitations of this review

An extensive literature search was carried out, with two reviewers screening full texts independently for inclusion and the data extracting independently checked. This minimised bias within this review process, however, no attempt was made to source unpublished studies, nor studies published in any other language than English. It is thought that identifying unpublished trials minimises publication bias [103]. However, this approach has been questioned by others, who suggest that truly unpublished trials frequently have poor methodology, and ones with better methodology often eventually become published [104]. It is not possible to know if the inclusion, if available, of any unpublished trials would considerably alter our conclusions, or if this truly is a weakness of this review.

### Comparison with other reviews

Our main findings differ very little from the 2008 review [13], however firmer conclusions about stabilisation exercises can be drawn from our review. In the 2008 review the majority of the studies favouring stabilisation exercises combined the exercises with some other form of treatment, implying that it was the package of care that was effective rather than stabilisation exercises alone. In our updated review the majority of the studies used stabilisation exercises as sole treatment, and as such the data synthesis looks more closely at stabilisation exercises as sole treatment.

Our findings were similar to the Wang et al. [80] review, which also concluded some short term benefit to pain and disability for stabilisation exercises over general exercise, with no long term benefit to pain. No comparison for long term follow-up for disability was made, and no attempt at analysing results against MCID was made. Of their five included articles we included three in our review. One was excluded for duplicating results from another included study, which was included in both of our and Wang et al’s reviews. It is therefore possible that their meta-analysis double counts these results [40, 79]. The other study we excluded during initial screening [105] looked at a relaxation yoga programme with meditation, chanting and counselling, and clearly doesn’t match our intervention definition. It is perhaps questionable that this study was included within Wang et al’s study [80].

Our findings differ from the Byström et al [22] review which concluded long term benefit to disability in favour of stabilisation exercises over general exercises and with regards to pain at intermediate term. The differences may be due to our inclusion of a further 15 publications; their inclusion of studies within the analysis with high risk of bias defined by means of a PEDro score of less than 6; or their use of a fixed effects meta-analysis model for pooled analysis where heterogeneity, as measured by *I*^2^, was less than 50%. Choosing fixed versus random effect models solely based upon the test for heterogeneity is considered incorrect, and should be made upon which model best fits the distribution of effects sizes [106]. We used a random effects model on all analyses, since there is inherent heterogeneity in low back pain within the literature. Using a fixed effects model incorrectly could over estimate the pooled effect sizes and underestimate the confidence interval width, thus reducing reliability of results [106].

## Conclusion

The results of this current systematic review suggest that stabilisation exercises improves low back pain symptoms, but no better than any other form of active exercise in the long term. The low levels of heterogeneity and large number of high methodological quality of available studies, at long term follow-up, strengthen our current findings. There is a trend of worse fear avoidance scores.

This review cannot recommend stabilisation exercises for low back pain in preference to other forms of general exercise, and further research is unlikely to considerably alter this conclusion.

## Electronic supplementary material

Additional file 1:Sensitivity analysis results.(XLSX 13 KB)

Below are the links to the authors’ original submitted files for images.

Authors’ original file for figure 1

Authors’ original file for figure 2

Authors’ original file for figure 3

Authors’ original file for figure 4

Authors’ original file for figure 5
