# Supplementary figures and images for: An update of stabilisation exercises for low back pain: a systematic review with meta-analysis
Source: BMC Musculoskelet Disord. 2014 Dec 9;15:416. doi: 10.1186/1471-2474-15-416 (PMC4295260; doi:10.1186/1471-2474-15-416)

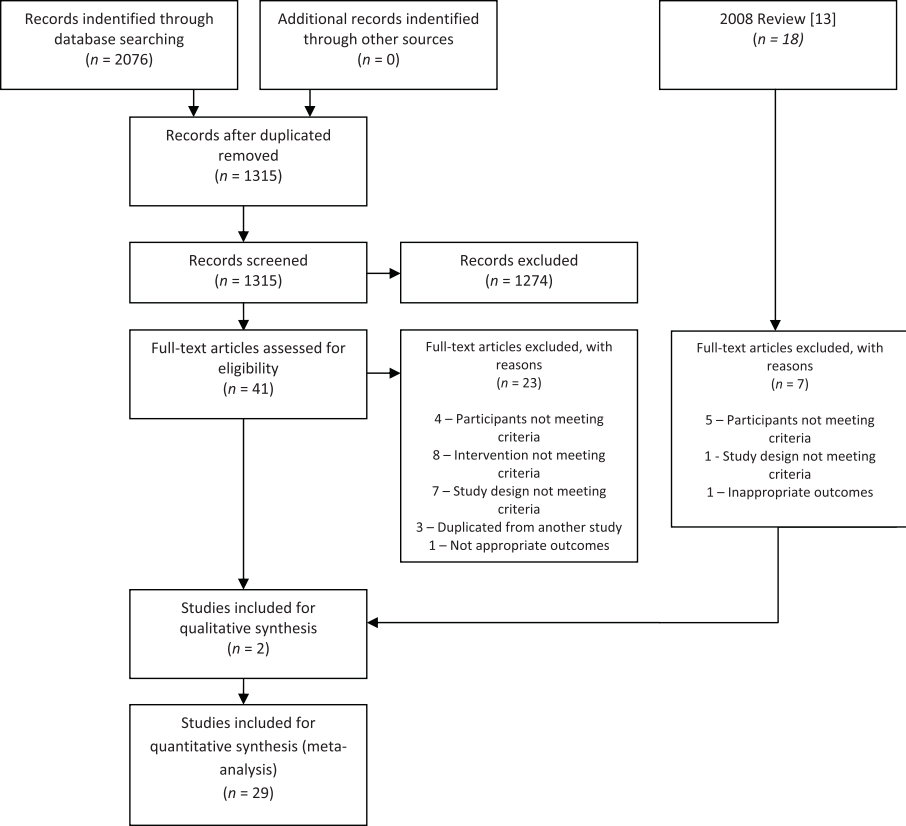

Supplement: Supplementary file 2 — Authors’ original file for figure 1 [file 12891_2014_2354_MOESM2_ESM.pdf]

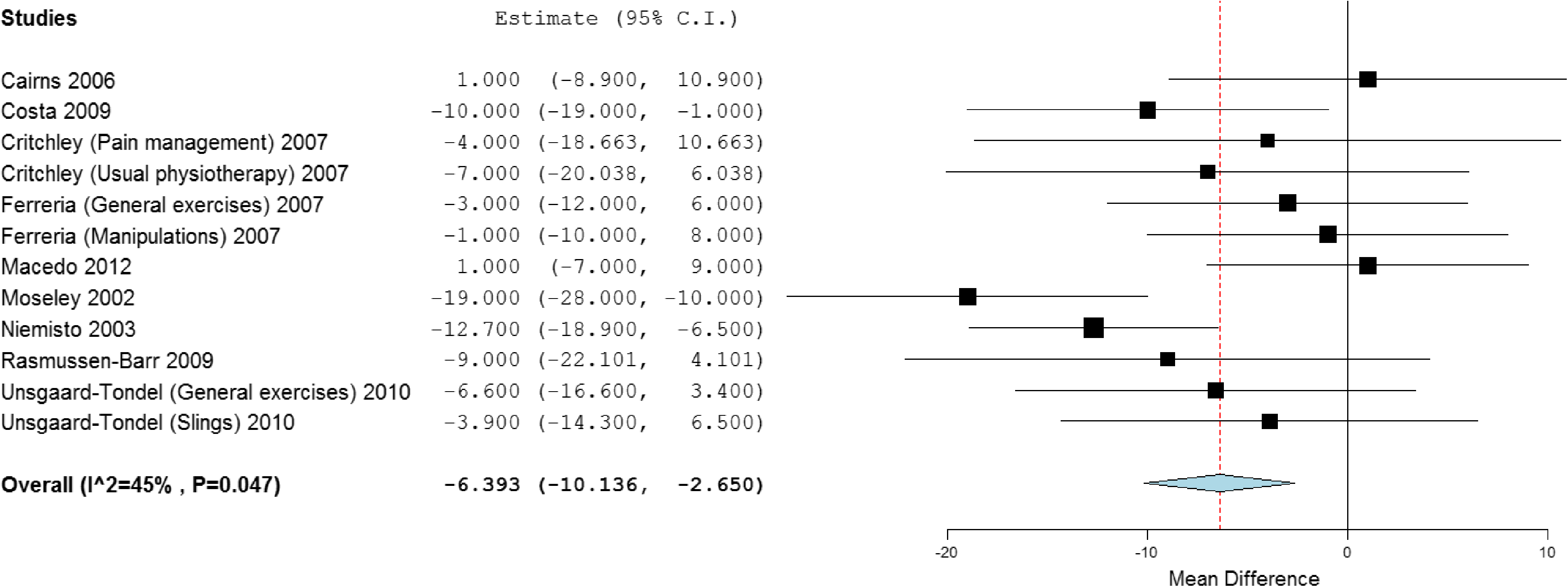

Supplement: Supplementary file 3 — Authors’ original file for figure 2 [file 12891_2014_2354_MOESM3_ESM.tif]

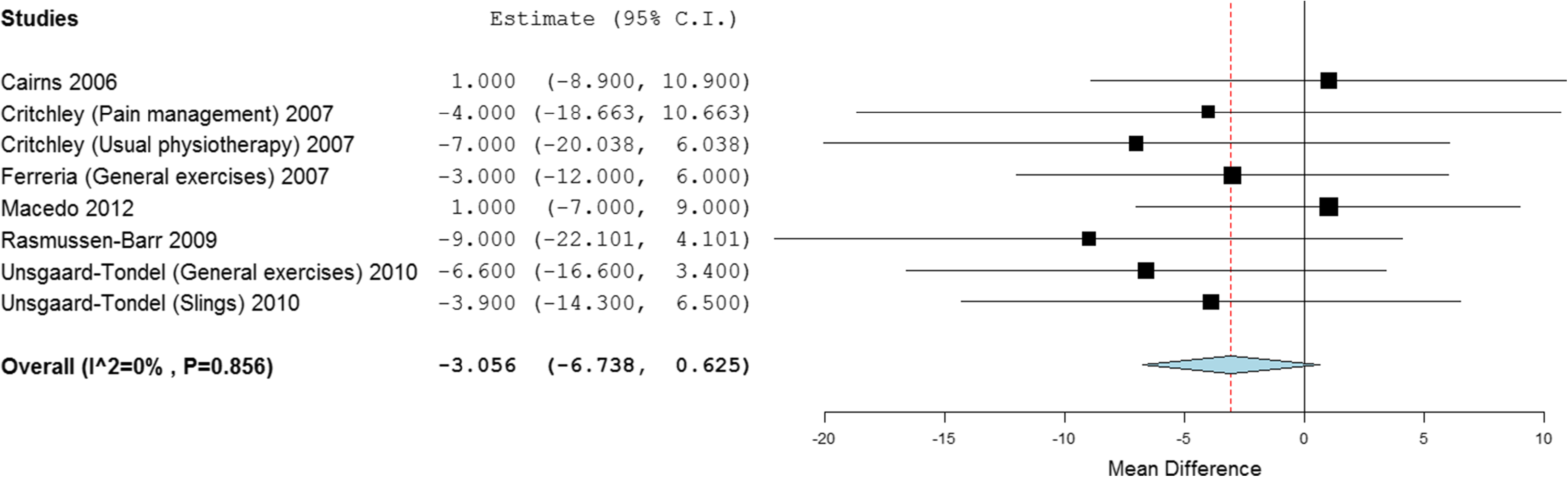

Supplement: Supplementary file 4 — Authors’ original file for figure 3 [file 12891_2014_2354_MOESM4_ESM.tif]

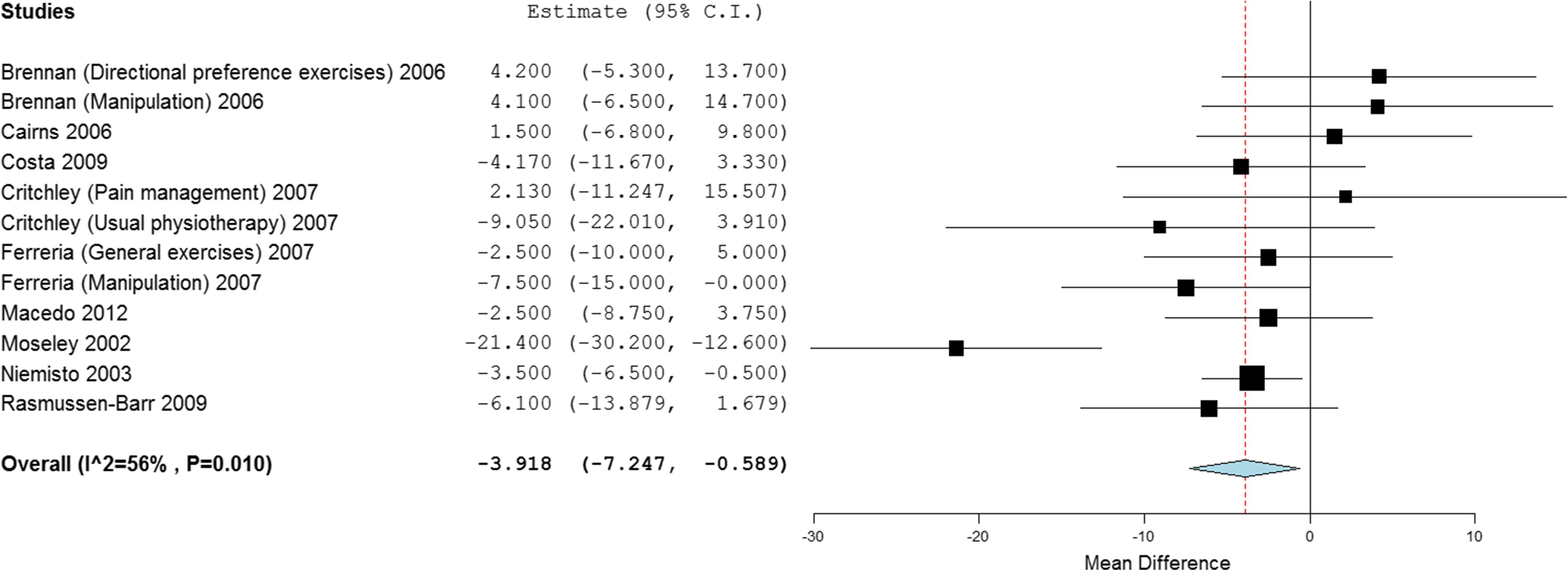

Supplement: Supplementary file 5 — Authors’ original file for figure 4 [file 12891_2014_2354_MOESM5_ESM.tif]

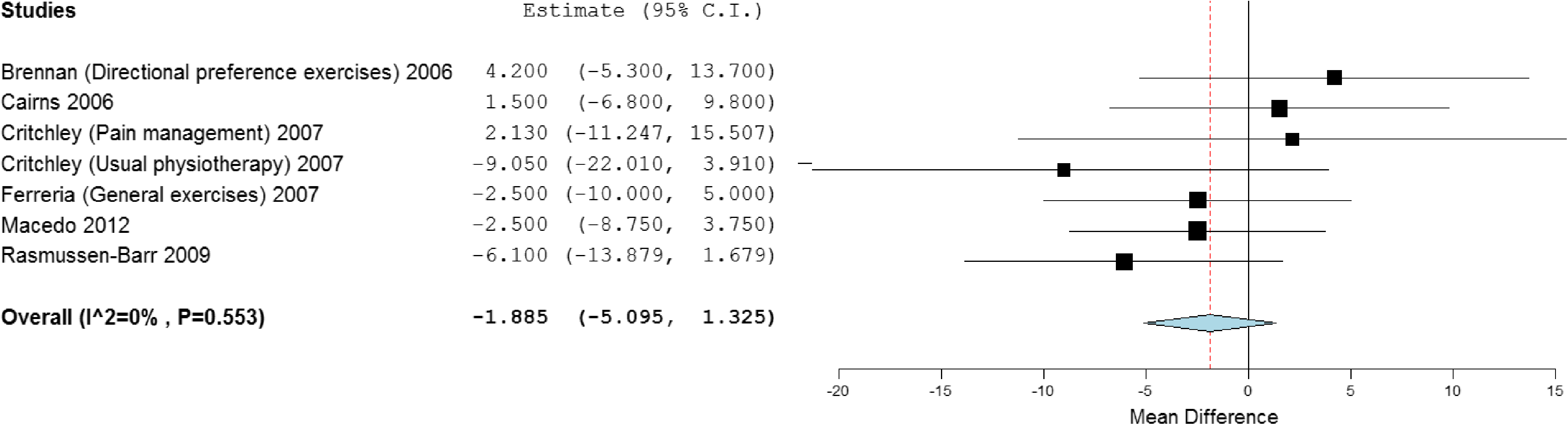

Supplement: Supplementary file 6 — Authors’ original file for figure 5 [file 12891_2014_2354_MOESM6_ESM.tif]
